# Management of Children With Food-Induced Anaphylaxis: A Cross-Sectional Survey of Parental Knowledge, Attitude, and Practices

**DOI:** 10.3389/fped.2022.886551

**Published:** 2022-05-19

**Authors:** Laura Polloni, Ileana Baldi, Margherita Amadi, Valentina Tonazzo, Roberta Bonaguro, Francesca Lazzarotto, Alice Toniolo, Dario Gregori, Antonella Muraro

**Affiliations:** ^1^Department of Women's and Children's Health, Food Allergy Referral Centre, Veneto Region, Padua University Hospital, Padua, Italy; ^2^Unit of Psychology, Padua University Hospital, Padua, Italy; ^3^Unit of Biostatistics, Epidemiology and Public Health, Department of Cardiac, Thoracic and Vascular Sciences and Public Health, University of Padua, Padua, Italy; ^4^Department of Women's and Children's Health, School of Medicine and Surgery, University of Padua, Padua, Italy

**Keywords:** food allergy, children, anaphylaxis, parents, adrenaline, autoinjector, management, education

## Abstract

**Background:**

Anaphylaxis is a life-threatening event, but it is frequently undertreated in pediatric patients with food allergies. Previous studies showed that auto-injectable adrenaline (AAI) is underused by patients and parents. This is especially troubling since fatal anaphylaxis has been associated with delayed adrenaline administration.

**Objectives:**

This study aimed to investigate parental practice and knowledge in anaphylaxis management, and perceived barriers and facilitators in using AAI.

**Results:**

A retrospective survey was completed by 75 parents (41 mothers, 34 fathers) of children with food allergy and AAI prescription attending the Food Allergy Referral Center of Veneto, Italy. Results showed poor parental preparedness and reluctance to use AAI despite a high/moderate self-rated knowledge (median *total score* of 23–min. 3, max. 30). Most parents (77%) declared they were carrying AAI but only 20% used it in case of a severe reaction. Most reported *Fear/Fear of making mistakes* (46 parents) and *Concern about possible side effects* as barriers (35), while *Poor knowledge of the correct AAI use* (1) and *Lack of knowledge/ incorrect assessment of symptoms* (2) were reported less frequently. *Theoretical-practical courses for parents on AAI use (65), Psycho-education/Psychological support* (3) for better dealing with the emotional aspects of anaphylaxis and *Written instructions* (1) have been suggested as main facilitators.

**Conclusion:**

Understanding parents' experience and perspective on managing anaphylaxis is crucial to implement effective educational programs. A multidisciplinary approach should be considered.

## Introduction

Anaphylaxis is a life-threatening reaction characterized by acute onset of symptoms involving different organ systems and requiring immediate intervention (4–6). Although the fatality rate due to anaphylaxis remains low (7), the frequency of hospitalization from food-induced anaphylaxis has been increasing in recent years (8). The symptoms of anaphylaxis are highly variable. Skin, mucosal and gastrointestinal symptoms occur most frequently (>90% of cases) followed by symptoms involving the respiratory and cardiovascular systems (>50%) (5, 6). Food is the most common elicitor of anaphylactic reactions among children and adolescents (9, 10). Intramuscular adrenaline is the treatment of choice for food-related anaphylactic reactions and adrenaline autoinjectors (AAI) are recommended for the first-line management of anaphylaxis in the community, since they are relatively safe, have a low risk of error and are fast to administer (5, 6). Although AAI are routinely prescribed for patients at risk of serious reactions, previous studies have shown an underuse by patients and parents (11–20). A retrospective study on children with a history of anaphylaxis (15) found that recurrent generalized allergic reactions occurred with a frequency of 0.98 episodes per patient per year and were more common in those with food compared with insect venom anaphylaxis. The AAI was only used in 29% of recurrent anaphylactic reactions. Parental knowledge was deficient in recognition of the symptoms of anaphylaxis and use of the AAI device. Those children in whom the AAI was used were less likely to require hospital admission (15).

Another study on parental use of AAI for children with food-induced anaphylaxis reported that only 8% of parents had administered it. Neither a history of anaphylaxis nor knowledge correlated with an increased level of comfort with administration (16). Similarly, a further research showed that 69% of parents were unable to use the AAI, did not have it available, or did not know when it should be administered (12).

This pattern is worrying given that most cases of reported deaths from anaphylaxis have been associated with delayed administration of epinephrine (13, 21). It is essential to understand the parents' perspective in order to facilitate the use of AAI. The present study is part of a larger project on quality of life and management of FA and anaphylaxis in the family. This preliminary part investigated parental previous experience of managing food-induced anaphylaxis, perceived knowledge, and perceived barriers and facilities about the use of AAI focusing mainly on descriptive and qualitative analysis of parents' viewpoint.

## Methods

### Participants

A total of 75 Italian parents of children with FA took part in the study. Inclusion criteria comprised that (4) children were confirmed suffering from immunoglobulin E (IgE)-mediated food allergy by an allergist considering their clinical history, the evidence of sensitization and a positive food challenge or positive skin prick test and/or serum-specific IgE results; (5) children were prescribed with an AAI.

All participants developed food allergy in early childhood and did not suffer from serious concomitant non-allergic disease. Food involved in patients' food allergy were milk, egg, wheat, fish and nuts.

Parents have been trained by an allergist to use AAI at least once a year through practical demonstration with an AAI training device and verbal instructions. They also received a written emergency plan for the school.

### Procedure

A survey about parental management and perceived knowledge of anaphylaxis was carried out at the Food Allergy Referral Center, Veneto Region in Padua (North Eastern Italy). Parents were invited to participate (as part of a larger project on quality of life and management of FA and anaphylaxis in the family) while accompanying the children to clinical visits. They were given an information sheet outlining the study and encouraged to ask the researcher questions. If interested in participating, they signed a written consent form. Participants were recruited sequentially over a period of 12 months. The project was approved by the Ethic Committee of Psychological Research of Padua University (registration number 7A30FE81F8F234480D5A94C49C270FF3). It was performed in respect of the European regulation regarding potential sensitive data and according to World Medical Association Declaration of Helsinki Ethical Principles for Medical Research Involving Human Subjects.

### Instruments

**Figure 1:**
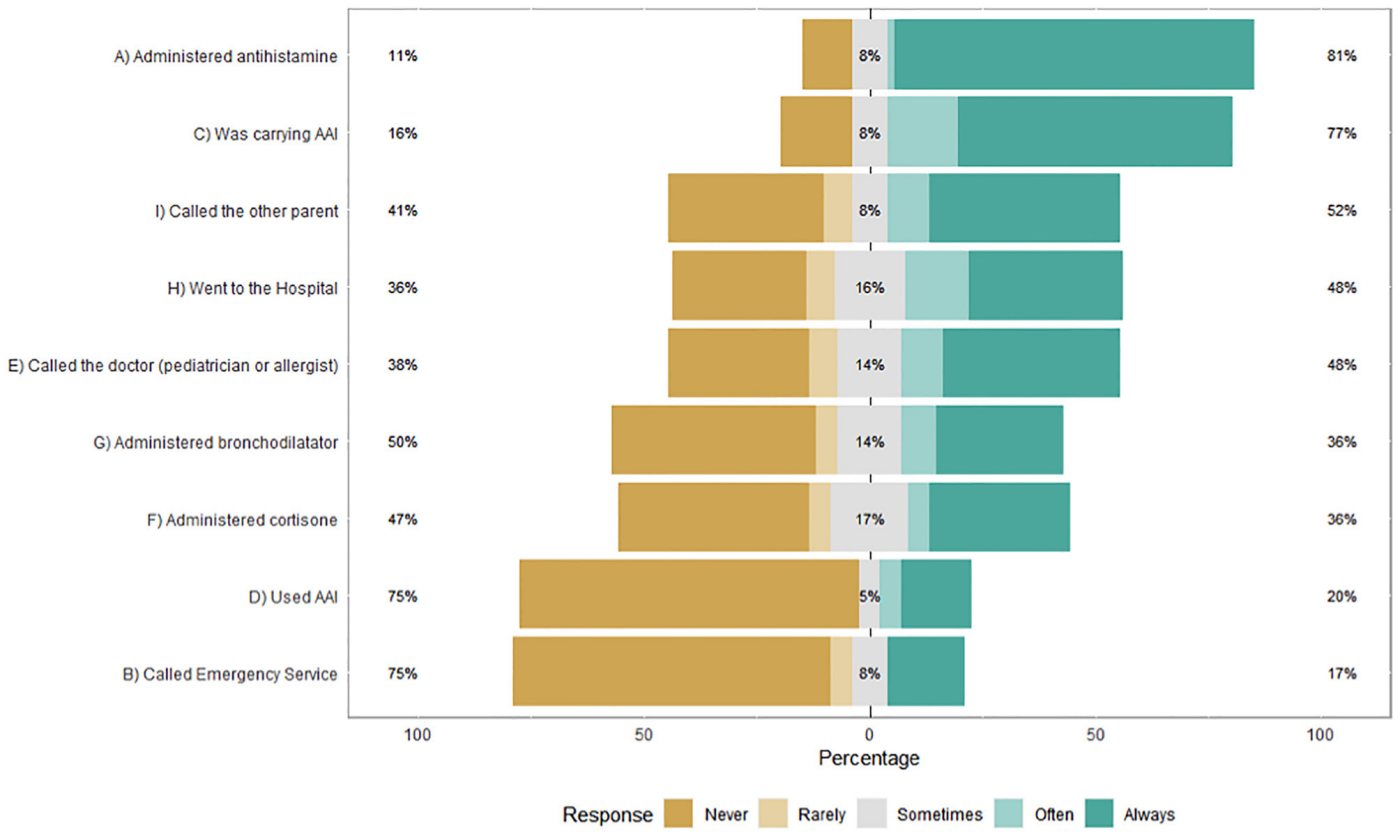
Parental responses on previous management of their child's anaphylaxis (*N* = 64).

The survey was made up of four sections:

1. Socio-demographic and clinical data: Clinical data reported by parents were cross-checked with medical record data.
2. Questions on previous anaphylactic experience: Parents were asked if they had ever managed an anaphylactic reaction (4–6) of their child, and if so, they were asked how they acted, choosing from a list of possible behaviors (showed in Figure 1). This list was devised following a focus group with some parents on how they behaved when managing a severe allergic reaction and based on current European guidelines on anaphylaxis management (5).
3. Parental self-rated knowledge on anaphylaxis management: Parents were asked to answer the following questions on a Likert scale from 1 (completely insufficient) to 10 (excellent)
  - How they defined their degree of knowledge of food-induced anaphylaxis (e.g., knowledge of symptoms)
  - How did they define their degree of knowledge of the correct use of AAI (e.g., how the AAI is used in practice)
  - How did they define their degree of knowledge of the management of anaphylaxis (e.g., what to do in case of anaphylaxis).
4. Questions on barriers and facilities about AAI parental use: Mothers and fathers were asked why, in their opinion, parents may be reluctant to administer AAI (barriers) and what could help them (facilities). Both questions were open-ended.

Parents reported having managed at least one of their child's anaphylaxis were asked to complete all the four sections of the survey. Parents who never managed their child's anaphylaxis completed sections 1, 3, and 4.

### Statistical Analysis

Descriptive analysis is presented stratified by parent's gender and overall, with percentages (number of cases) or median (first, third quartile), as appropriate. Fisher and Chi square test were used for univariable analysis. A linear regression model was used to investigate the effect of socio-demographic characteristics and previous anaphylactic experience on perceived knowledge total score. The model building strategy was established on the basis of a backward selection procedure and the Akaike Information Criterion (AIC). The concept underlying the AIC is to examine the complexity of the model together with its goodness-of-fit to the sample data and to produce a measure balancing the two. The usual procedure is to compute the AIC for each model and select that corresponding to the lowest value.

In addition, a qualitative analysis was carried out on parents' open responses about the barriers and facilitators of using AAI. The analysis of the texts was carried out by two independent judges (MA and VT) who identified and attributed categories that could describe parental responses in a salient way. The identified categories were supervised by LP and AM. In the phase of attribution of the categories, in case of lack of agreement between the judges, the category was discussed until 100% agreement was reached.

## Results

Parents and children's characteristics are shown in Table 1. Thirty-four fathers and 41 mothers of children with FA and AAI prescription (age 3–14) participated. Most children had multiple FA (69.3%) and comorbidity with asthma and/or eczema (77.3%). Sixty-four parents (85.3%) reported having managed at least one of their child's anaphylaxis and fully completed the second section of the survey (*Questions on previous anaphylactic experience*), while the remaining parents completed only section 1, 3, and 4. As showed in Figure 1, 77% of parents were carrying AAI and 20% would have used AAI often or always. Most parents, 81%, administered antihistamines, 52% asked the other parent for help, 48% went to the hospital or called the doctor, 35% administered bronchodilator and 36% cortisone, while 17% called the emergency service. No differences appear when the responses are stratified by gender (Supplementary Table 1).

**Table 1:** Participants' characteristics stratified by parent's gender and overall, with percentages (number of cases) or median (first, third quartile), as appropriate.

	***N***	**Male** **(*N* = 34)**	**Female** **(*N* = 41)**	**Combined** **(*N* = 75)**
**Parents age**	75	43.0/46.0/48.0	40.0/43.0/47.0	41.0/45.0/47.5
**Parents education**	74			
Lower secondary		11.8% (7)	15.0% (9)	13.5% (13)
Upper secondary		73.5% (22)	45.0% (21)	58.1% (43)
Higher		14.7% (8)	40.0% (19)	28.4% (1)
**Job**	75			
Stay-at-home parent		0.0% (0)	26.8% (14)	14.7% (14)
Clerk		20.6% (10)	2.4% (4)	10.7% (11)
Employee		41.2% (17)	56.1% (3)	49.3% (37)
Self-Employed		32.4% (14)	9.8% (7)	20.0% (18)
Manager		5.9% (5)	4.9% (5)	5.3% (7)
**Child sex**	75			
F		50.0% (20)	56.1% (3)	53.3% (40)
**Child age**	75	7/ 9/12	7/10/12	7/ 9/12
**Nr of food allergies**	75			
1		20.6% (10)	17.1% (10)	18.7% (17)
2		11.8% (7)	12.2% (8)	12.0% (12)
>2		67.6% (3)	70.7% (23)	69.3% (52)
**Comorbidity with asthma/eczema**	75			
Yes		79,4% (24)	75.6% (31)	77.3% (58)
**Parental experience of managing child's anaphylaxis (nr of episodes)**	75			
0		17.6% (9)	12.2% (8)	14.7% (14)
1		29.4% (13)	26.8% (14)	28.0% (1)
>1		52.9% (21)	61.0% (22)	57.3% (43)

With regard to *Parental self-rated knowledge on anaphylaxis management*, parents reported a median score of 7 on a 1–10 Likert scale on *knowledge of anaphylaxis symptoms*, a median score of 8 on *knowledge of AAI Use*, a median score of 7 on *knowledge of anaphylaxis management* and a median *total score* of 23 (min. 3, max. 30). The Fisher test found no significant differences between mothers and fathers' scores (Supplementary Table 2).

Stepwise regression results (Table 2) indicate that one age increase in patient age and previous episodes of managing anaphylaxis (as compared to none) significantly increase the mean Total score of 0.53 and 3.15, respectively.

**Table 2:** Linear regression on perceived knowledge total score with backward selection of variables based on AIC.

	**Coefficient [95% CI]**	***p*-value**
Patient age	0.53 [0.26, 0.80]	<0.01
Comorbidity with asthma/eczema (yes vs. no)	−2.49 [−5.52, 0.54]	0.11
Parental experience of managing child's anaphylaxis (nr of episodes) (1 vs. 0)	1.46 [−1.84, 4.76]	0.38
Parental experience of managing child's anaphylaxis (nr of episodes) (>1 vs. 0)	3.15 [0.16, 6.14]	0.04

As showed in Table 3 qualitative analysis on parents' open responses about the barriers and facilitators of using AAI led to the identification of four categories for each. The barriers identified by the parents are in order of frequency: (4) *Fear/Fear of making mistakes;* (5) *Concern about possible side effects resulting from the administration of adrenaline;* (6) *Poor knowledge of the correct procedure for using the AAI device;* (7) *Lack of knowledge/ incorrect assessment of symptoms that require AAI*. The facilitators identified in order of frequency are: (4) *Theoretical-practical courses on AAI use for parents;* (5) *Psycho-education/Psychological support;* (6) *Written Instructions;* (7) *Parent support groups*. More than one answer was possible.

**Table 3:** Qualitative analysis on parents' open responses about the barriers and facilitators of using AAI: categories, frequencies, and examples.

**Questions**	**Response categories, frequencies, and examples**			
**Barriers**	**Fear/Fear of making mistakes**	**Concern about possible side effects resulting from the administration of adrenaline**	**Poor knowledge of the correct use of the AAI device**	**Lack of knowledge/incorrect assessment of symptoms that requireAAI**
Why parents may be reluctant to administer AAI?	46	35	21	19
Examples of answers	Because it's scary (Mother of a 13-year-old girl) For fear of making mistakes and making the situation worse (Mother of a 13-year-old girl) Fear of making mistakes, of doing wrong (Mother of a 5-year-old boy)	They don't know the reaction that can occur after the injection (Father of a 10-year-old boy) I think they are afraid of side effects. (Mother of a 10-year-old boy). For fear of administering adrenaline when it is not necessary, causing negative physical and psychological consequences. (Father of a 11-year-old girl)	Fear of not being able to inject it correctly (Mother of a 6-year-old boy) They do not remember how to inject adrenaline even if it has been explained several times... (Mother of a 13-year-old boy) Not optimal knowledge of the procedures (Father of a 10-year-old boy)	Fear of being reckless and proceeding with a procedure not suitable for the case (Father of a 13-year-old girl) Fear of not being able to understand if the reaction requires the use of adrenaline (Father of a 12-year-old girl) Not having idea of when is the right moment to administer adrenaline (Father of a 4-year-old boy)
**Facilitators**	**Theoretical-practical courses on AAI use for parents**	**Psychoeducation/Psychological support**	**Written Instructions**	**Parent support groups**
What could help parents?	65	23	21	6
Examples of answers	Specific courses, mainly based on practical demonstrations (father of a 14-year-old girl) After the first diagnosis of allergy, an ad-hoc course should be arranged for the parents (father of a 3-year-old boy) Practical training, such as practicing with a mannequin (father of a 11-year-old boy	Psychological support can help to better cope and deal with an emergency (mother of a 11-year-old girl) Psychological support can be really helpful (mother of a 12-year-old girl) Psychological support is essential (father of a 7-year-old boy)	Written instructions reporting all possible symptoms, from the most to the least severe (mother of a 9-year-old boy) Always have printed instructions with sample photos at hand (father of a 14-year-old girl) Instructions on how to use the AAI and when, recognize the symptoms, etc. (mother of a 7-year-old girl)	Meetings with other parents who have had a similar experience (father of a 7-year-old girl) Talk with other parents who have experienced and are experiencing the same difficulties (mother of a 11-year-old girl) Testimonials (mother of a 5-year-old boy)

## Discussion

The results of this survey showed poor parental preparedness for managing food-induced anaphylaxis and reluctance to use AAI despite a routine training (at least once a year) and a high/moderate self-rated knowledge on anaphylaxis management. The majority of parents (81%) administered antihistamines, and 36% cortisone, although they are both not recommended for the acute management of anaphylaxis (2). If used prior to adrenaline administration, antihistamines/glucocorticoids administration could lead to a delay in first-line treatment of anaphylaxis. Adrenaline is the first-line treatment of anaphylaxis because it has a faster onset of action and more appropriate and robust pharmacologic action (2, 6, 10). Most parents (77%) declared they were carrying AAI at the time of their children severe allergic reactions but only 20% would have used it often or always. Results are in line with previous researches (11–19) and support the importance of investigating the reasons why parents do not act properly in case of anaphylaxis. A recent study involving 164 caregivers (17) reported that the majority did not give their allergic children AAI at the time of their most severe allergic reactions, despite declaring confidence in their ability to treat allergic reactions. Reasons caregivers indicated for not administering the AAI included the following: reactions did not seem severe enough; use of other medication; and fear of using AAI. A qualitative study (ten parents involved) exploring parental experiences with AAI use for their child's anaphylaxis found that parents uniformly described feelings of isolation, fear/anxiety, hesitation and guilt during reactions, that constitute emotional barriers. Accordingly, parents in the present study mostly reported *Fear/Fear of making mistakes* and *Concern about possible side effects* as barriers, while *Poor knowledge of the correct AAI use* and *Lack of knowledge/ incorrect assessment of symptoms* were reported less frequently. A number of previous studies on parental preparedness for managing anaphylaxis focused on knowledge (e.g., recognition of the symptoms of anaphylaxis and use of the AAI device) (1, 12, 15, 25) highlighting specific knowledge deficits that could impact negatively. In addition to deficiencies in knowledge, given the critical nature of anaphylaxis, the psychological component (e.g., fear or anxiety) has been found preponderant to the underuse of AAI (3, 16). A study involving 165 parents reported that parental empowerment directly correlated with increased comfort with AAI use, but knowledge did not (16). Other researches (3, 11, 17) found that the most common reasons among parents for not using adrenaline despite carrying was concern about adverse effects, fear about the injection, and not wanting to activate emergency services or go to the emergency department.

Fear might be a psychological factor that paralyzes instead of enabling a parent to act accordingly in the event of anaphylaxis in their child (3, 16). Prior studies (22, 24, 26–28) have confirmed the psychological effect that food allergy has on families functioning and wellbeing. These burdens might also impair a parent's response to an acute life-threatening event. It has been demonstrated that among parents who experienced their child anaphylaxis, 14.6% developed an acute stress disorder and this was comparable to the percentage of PTSD in adults who experienced anaphylaxis themselves (23). Further researches should investigate psychological factors (e.g., fear/anxiety, self-efficacy) contributing to parental preparedness for managing food-induced anaphylaxis in order to remove or mitigate barriers.

To our knowledge this is the first study exploring parental perspective not only about barriers but also facilitators. Parents reported *Theoretical-practical courses on AAI use for parents* as main help and *Written available instructions*. This underscores the need for continued focus on educating patients and caregivers to recognize signs and symptoms of anaphylaxis, understanding the role of different allergy medications and effectively using AAI (17). Many participants suggested *Psycho-education/Psychological support* for better dealing with the emotional aspects of anaphylaxis management (e.g., fear/anxiety, stress) and a limited number reported *Parent support groups* to share experiences. This highlights the importance to develop effective training strategies to prepare caregivers to act during stressful situations. It is worth to note that the Food Allergy Center of Veneto Region has a dedicated psychological service for supporting patients and families in the psycho-socio-emotional management of food allergy and anaphylaxis.

This preliminary study is very descriptive in its nature and some limitations need to be acknowledged. The sample is quite homogeneous in its characteristics and all patients were attending a third level Food Allergy Referral Center, which limits generalizability of the results. In addition, the retrospective design is subject to recall bias, as participants were asked to describe their child's anaphylaxis, which could have occurred months to years before the survey was completed. Despite these limitations, this study offers a unique overview on parents experience and perspective on managing food-induced anaphylaxis considering not only barriers but also facilitators. The psychological component seems to be crucial and need to be addressed. This is essential to develop novel interventions to improve food allergy management in pediatric patients. We previously confirmed that multidisciplinary training for anaphylaxis management in the school setting, that included theoretical, practical and psychological aspects, showed efficacy in improving management by school personnel (29). This should be implemented also for parents in order to improve safety and wellbeing of children with severe food allergy.

## Data Availability Statement

The original contributions presented in the study are included in the article/Supplementary Material, further inquiries can be directed to the corresponding author.

## Ethics Statement

The studies involving human participants were reviewed and approved by Ethic Committee of Psychological Research of Padua University. The patients/participants provided their written informed consent to participate in this study.

## Author Contributions

LP contributed to study design, data collection and interpretation, and writing of the draft of the manuscript. IB contributed to statistical design and analysis, data interpretation, and writing of the manuscript. MA and VT contributed to study design, data collection, data interpretation, and critically reviewed the manuscript. RB, FL, and AT contributed to study design, data interpretation, and critically reviewed the manuscript. DG contributed to statistical design and analysis, data interpretation, and critically reviewed the manuscript. AM contributed to study design, data interpretation, project administration, supervision, and critically reviewed the manuscript. All authors have read and agreed to the published version of the manuscript.

## Conflict of Interest

The authors declare that the research was conducted in the absence of any commercial or financial relationships that could be construed as a potential conflict of interest.

## Publisher's Note

All claims expressed in this article are solely those of the authors and do not necessarily represent those of their affiliated organizations, or those of the publisher, the editors and the reviewers. Any product that may be evaluated in this article, or claim that may be made by its manufacturer, is not guaranteed or endorsed by the publisher.
