# Supplementary material for: Management of Children With Food-Induced Anaphylaxis: A Cross-Sectional Survey of Parental Knowledge, Attitude, and Practices
Source: Front Pediatr. 2022 May 19;10:886551. doi: 10.3389/fped.2022.886551 (PMC9160827; doi:10.3389/fped.2022.886551)
Supplement: Supplementary file 1 [file Table_1.DOCX]

Table S1. Questionnaire on previous anaphylactic experience by parent’s gender. First quartile/median/third quartile and p-value (P) of Chi-square test with given degrees of freedom (d.f.).

|  | M (N=28) | F (N=36) | Combined (N=64) | Test Statistic |
| --- | --- | --- | --- | --- |
| **A) Administered Antihistamine** |  |  |  | Chi-square=7.62 d.f.=3 P=0.055 |
| **Never** | 17.9% (5) | 5.6% (2) | 10.9% (7) |  |
| **Rarely** | 0.0% (0) | 0.0% (0) | 0.0% (0) |  |
| **Sometimes** | 14.3% (4) | 2.8% (1) | 7.8% (5) |  |
| **Often** | 3.6% (1) | 0.0% (0) | 1.6% (1) |  |
| **Always** | 64.3% (18) | 91.7% (33) | 79.7% (51) |  |
| **B) Called Emergency Service** |  |  |  | Chi-square=0.92 d.f.=3 P=0.82 |
| **Never** | 71.4% (20) | 69.4% (25) | 70.3% (45) |  |
| **Rarely** | 3.6% (1) | 5.6% (2) | 4.7% (3) |  |
| **Sometimes** | 10.7% (3) | 5.6% (2) | 7.8% (5) |  |
| **Often** | 0.0% (0) | 0.0% (0) | 0.0% (0) |  |
| **Always** | 14.3% (4) | 19.4% (7) | 17.2% (11) |  |
| **C) Was Carrying AAI** |  |  |  | Chi-square=1.25 d.f.=3 P=0.742 |
| **Never** | 14.3% (4) | 16.7% (6) | 15.6% (10) |  |
| **Rarely** | 0.0% (0) | 0.0% (0) | 0.0% (0) |  |
| **Sometimes** | 7.1% (2) | 8.3% (3) | 7.8% (5) |  |
| **Often** | 10.7% (3) | 19.4% (7) | 15.6% (10) |  |
| **Always** | 67.9% (19) | 55.6% (20) | 60.9% (39) |  |
| **D) Used AAI** |  |  |  | Chi-square=0.83 d.f.=3 P=0.842 |
| **Never** | 75.0% (21) | 75.0% (27) | 75.0% (48) |  |
| **Rarely** | 0.0% (0) | 0.0% (0) | 0.0% (0) |  |
| **Sometimes** | 7.1% (2) | 2.8% (1) | 4.7% (3) |  |
| **Often** | 3.6% (1) | 5.6% (2) | 4.7% (3) |  |
| **Always** | 14.3% (4) | 16.7% (6) | 15.6% (10) |  |
| **E) Called The Doctor (Pediatrician Or Allergist)** |  |  |  | Chi-square=4.92 d.f.=4 P=0.296 |
| **Never** | 35.7% (10) | 27.8% (10) | 31.2% (20) |  |
| **Rarely** | 7.1% (2) | 5.6% (2) | 6.2% (4) |  |
| **Sometimes** | 21.4% (6) | 8.3% (3) | 14.1% (9) |  |
| **Often** | 10.7% (3) | 8.3% (3) | 9.4% (6) |  |
| **Always** | 25.0% (7) | 50.0% (18) | 39.1% (25) |  |
| **F) Administered Cortisone** |  |  |  | Chi-square=7.39 d.f.=4 P=0.117 |
| **Never** | 39.3% (11) | 44.4% (16) | 42.2% (27) |  |
| **Rarely** | 10.7% (3) | 0.0% (0) | 4.7% (3) |  |
| **Sometimes** | 25.0% (7) | 11.1% (4) | 17.2% (11) |  |
| **Often** | 3.6% (1) | 5.6% (2) | 4.7% (3) |  |
| **Always** | 21.4% (6) | 38.9% (14) | 31.2% (20) |  |
| **G) Administered Bronchodilatator** |  |  |  | Chi-square=0.74 d.f.=4 P=0.946 |
| **Never** | 42.9% (12) | 47.2% (17) | 45.3% (29) |  |
| **Rarely** | 3.6% (1) | 5.6% (2) | 4.7% (3) |  |
| **Sometimes** | 17.9% (5) | 11.1% (4) | 14.1% (9) |  |
| **Often** | 7.1% (2) | 8.3% (3) | 7.8% (5) |  |
| **Always** | 28.6% (8) | 27.8% (10) | 28.1% (18) |  |
| **H) Went To The Hospital** |  |  |  | Chi-square=3.72 d.f.=4 P=0.445 |
| **Never** | 25.0% (7) | 33.3% (12) | 29.7% (19) |  |
| **Rarely** | 7.1% (2) | 5.6% (2) | 6.2% (4) |  |
| **Sometimes** | 25.0% (7) | 8.3% (3) | 15.6% (10) |  |
| **Often** | 14.3% (4) | 13.9% (5) | 14.1% (9) |  |
| **Always** | 28.6% (8) | 38.9% (14) | 34.4% (22) |  |
| **I) Called The Other Parent** |  |  |  | Chi-square=0.39 d.f.=4 P=0.983 |
| **Never** | 35.7% (10) | 33.3% (12) | 34.4% (22) |  |
| **Rarely** | 7.1% (2) | 5.6% (2) | 6.2% (4) |  |
| **Sometimes** | 7.1% (2) | 8.3% (3) | 7.8% (5) |  |
| **Often** | 7.1% (2) | 11.1% (4) | 9.4% (6) |  |
| **Always** | 42.9% (12) | 41.7% (15) | 42.2% (27) |  |
