# Supplementary material for: Management of Children With Food-Induced Anaphylaxis: A Cross-Sectional Survey of Parental Knowledge, Attitude, and Practices
Source: Front Pediatr. 2022 May 19;10:886551. doi: 10.3389/fped.2022.886551 (PMC9160827; doi:10.3389/fped.2022.886551)
Supplement: Supplementary file 2 [file Table_2.DOCX]

Table S2. Parental self-rated knowledge on anaphylaxis management by parent’s gender. First quartile/median/third quartile and p-value (P) of Fisher (F) test with given degrees of freedom (d.f.).

|  | M  (N=34) | F (N=41) | Combined (N=75) | Test Statistic |
| --- | --- | --- | --- | --- |
| **1) Knowledge of Anaphylaxis Symptoms** | 6/7/8 | 7/8/9 | 6/7/8 | F=2.25 d.f.=1,73 P=0.138 |
| **2) Knowledge of AAI Use** | 7/8/9 | 7/8/9 | 7/8/9 | F=0.24 d.f.=1,73 P=0.629 |
| **3) Knowledge of Anaphylaxis Management** | 6/8/8.75 | 7/7/9 | 7/7/9 | F=0.18 d.f.=1,73 P=0.671 |
| **Total Score** | 19/22.5/25.75 | 20/23/26 | 19.5/23/26 | F=0.49 d.f.=1,73 P=0.488 |
